# Supplementary material for: Tracking ℛ of COVID-19: A new real-time estimation using the Kalman filter
Source: PLoS One. 2021 Jan 13;16(1):e0244474. doi: 10.1371/journal.pone.0244474 (PMC7806155; doi:10.1371/journal.pone.0244474)
Supplement: S1 Appendix — Supplementary appendix containing additional theoretical and simulation results, data descriptions, and further empirical analysis. (PDF) [file pone.0244474.s001.pdf]

Supplementary Appendix for: "Tracking $\mathcal{R}$ of COVID-19: A New Real-Time Estimation Using the Kalman Filter"

Francisco Arroyo-Marioli[1], Francisco Bullano[1], Simas Kučinskas*[2], and Carlos Rondón-Moreno[1]

[1]*Central Bank of Chile*
[2]*Humboldt University of Berlin*

A.1 SIS Model

We now show that the estimator in the main text in Eq. (3) is also obtained when the dynamics of the disease follow the SIS model. The SIS model, again in discrete time, is given by

$$S_t = S_{t-1} - \beta_t I_{t-1} \frac{S_{t-1}}{N} + \gamma I_{t-1}$$
$$I_t = I_{t-1} + \beta_t I_{t-1} \frac{S_{t-1}}{N} - \gamma I_{t-1}$$

The only difference from the SIR model in the main text in Eq. (1) is that formerly infected individuals do not obtain immunity after recovery and instead again join the pool of susceptibles. As is well known, the basic reproduction number in the SIS model is the same as in the SIR model (e.g., Chowell and Brauer, 2009) and given by $\mathcal{R}_0^{(t)} = \beta_t/\gamma$. Since the law of motion for I_t in the SIS model is the same as in the SIR model, we can repeat the same steps as in the benchmark analysis to arrive at the same estimator as in in Eq. (3).

*Corresponding author: simas.kucinskas@hu-berlin.de.

A.2 Generalized SIR Model

In this section, we show that we can also obtain the estimator in Eq. (3) from a generalized version of the SIR model with stochastic shocks. Specifically, we consider the following generalized SIR model:

$$
\begin{aligned}
S_t &= S_{t-1} - \beta_t I_{t-1} \frac{S_{t-1}}{N} - v_{1,t} \\
I_t &= I_{t-1} + \beta_t I_{t-1} \frac{S_{t-1}}{N} - \gamma I_{t-1} + v_{1,t} - v_{2,t} \\
R_t &= R_{t-1} + \gamma I_{t-1} + v_{2,t}
\end{aligned}
$$

Differently from the baseline model, we introduce random shocks $v_{1,t}$ and $v_{2,t}$. The shocks are i.i.d., and the time-varying support of $v_{1,t}$ is $[0, S_{t-1} - \beta_t I_{t-1}/N]$, while the support of $v_{2,t}$ is $[0, I_{t-1} + \beta_t I_{t-1} S_{t-1}/N]$. We also assume that $\mathbb{E}_{t-1}[v_{1,t} - v_{2,t}] = 0$, so that the conditional expectation $\mathbb{E}_{t-1}[I_t]$ coincides with the value for I_t given by the noiseless SIR model. With these modifications, the model can capture rich patterns of infectious disease dynamics. For example, "super spreader events" can be modeled either as $v_{1,t}$ shocks or as a spike in β_t. The model can also capture richer forms of population structures than the baseline SIR model. For example, if individuals who are more infectious (e.g., those with more connections in a network model) are more likely to become infected first, that can be captured by assuming that β_t becomes lower over time.

Defining the time-varying basic reproduction number as $\mathcal{R}_0^{(t)} = \beta_t/\gamma$, and $\mathcal{R}_t \equiv \mathcal{R}_0^{(t)} S_{t-1}/N$, we obtain that

$$
\text{gr}(I_t) = \gamma(\mathcal{R}_t - 1) + v_t,
$$

where $v_t \equiv (v_{1,t} - v_{2,t})/I_{t-1}$. Taking expectations on both sides of the equation, we arrive at

$$
\mathbb{E}[\mathcal{R}_t] = 1 + \frac{1}{\gamma}\,\mathbb{E}[\text{gr}(I_t)].
$$

Hence, the generalized SIR model of the present section leads to the same estimator as the baseline SIR model.

Finally, we note that if γ varies deterministically over time, the equation above remains essentially unchanged, the only difference being that γ is replaced by γ_t. If γ_t follows a non-degenerate stochastic process, then the estimator for $\mathbb{E}[\mathcal{R}_t]$ would need to correct for the covariance between γ_t and $\mathcal{R}_t$.

A.3 Foundation for the Local-Level Model

When estimating $\mathcal{R}_t$, we use a local-level specification for the growth rate of the number of infected individuals. In this section, we show that the local-level model arises naturally in the SIR model in the early stages of an epidemic when the transmission rate follows a random walk.

Specifically, consider the generalized SIR model in Section A.2 of the Supplementary Appendix. We now specialize the process for the transmission rate β_t to be a random walk:

$$\beta_t = \beta_{t-1} + \eta_t, \eta_t \sim \text{i.i.d. } \mathcal{N}(0, \sigma_\eta^2),$$

with a given initial value $\beta_0 > 0$. We calculate that

$$\text{gr}(I_t) = \left(\frac{S_{t-1}}{N}\right)\beta_t - \gamma + v_t \approx \beta_t - \gamma + v_t$$

in the early stages of the epidemic when $S_t \approx N$. Defining the effective reproduction number early on in the epidemic as $\mathcal{R}_t = \beta_t/\gamma$, we therefore have directly that the growth rate of I_t follows a local-level model with

$$\begin{aligned} \text{gr}(I_t) &= \gamma(\mathcal{R}_t - 1) + v_t \\ R_t &= R_{t-1} + \tilde{\eta}_t \end{aligned}$$

where $\tilde{\eta}_t \equiv \eta_t/\gamma$. Provided that the distribution of v_t can be approximated with a normal distribution, we directly obtain the specification in the main text in Eq. (5). Alternatively, to obtain an exact normal local-level model, we could assume that $v_{1,t} = v_{2,t} = 0$ (no shocks in the original model, just as in the baseline model in the main text in Eq. (1)) but that instead of observing the true growth rate $\text{gr}(I_t)$, we only observe $\text{gr}(I_t) + \varepsilon_t$ where ε_t is i.i.d. normally distributed mean-zero measurement error.

A.4 Gibbs-Sampling Algorithm

In this section we discuss how the parameters of the state-space model can be estimated with a Gibbs-sampling algorithm *à la* Carter and Kohn (1994). Besides being a natural robustness check to our methodology, this algorithm uses a different approach to ensure the non-negativity of $\mathcal{R}_t$.

To use the Kalman filter, we need to estimate σ_ϵ^2 and σ_η^2 in the state-space model in Eq. (5) of the main text. For the Gibbs sampler, we break the model down into conditional densities from which we can sample iteratively. The algorithm is the following:

1. Conditional on σ_ε^2 and σ_η^2, use the Kalman filter to infer the state vector $\mathcal{R}_t$;
2. Conditional on the sequence of $\mathcal{R}_t$ computed in the previous step, take samples of σ_ε^2 and σ_η^2 from their prior distributions;
3. Conditional on the new draws of σ_ε^2 and σ_η^2, estimate $\mathcal{R}_t$;
4. Verify that each element of $\mathcal{R}_t$ is positive. If yes, store the draws of σ_ε^2 and σ_η^2. If not, discard the draws and repeat step 2;
5. Compute the Kalman smoother;
6. Iterate forward for as many replications as needed.

Finally, we contrast the estimates of $\mathcal{R}_t$ obtained with the Gibbs sampler to our baseline estimates. We obtain a correlation of 0.85 between the two sets of estimates. Credible intervals are highly correlated as well. Overall, we conclude that our estimates are similar across different statistical estimation approaches.

A.5 SEIR Model: Monte Carlo Simulation

Our estimation method uses a structural mapping between $\mathcal{R}_t$ and $\text{gr}(I_t)$ derived from the basic SIR model. While we can generalize the baseline SIR model to include stochastic shocks (Section A.2 of the Supplementary Appendix), and the estimator remains valid when the disease follows an SIS model (Section A.1 of the Supplementary Appendix), the model is nevertheless restrictive. In particular, it ignores incubation periods as well as transmission during the incubation period. These features are likely especially important when modeling COVID-19.

We now perform a simulation exercise to see how our estimator of $\mathcal{R}_t$ performs in a richer model that accounts for these additional features. Specifically, we consider an SEIR model in which the exposed are infectious:

$$\begin{aligned}
S_t &= S_{t-1} - \beta I_{t-1}\frac{S_{t-1}}{N} - \beta\epsilon E_{t-1}\frac{S_{t-1}}{N} \\
E_t &= E_{t-1} + \beta I_{t-1}\frac{S_{t-1}}{N} + \beta\epsilon E_{t-1}\frac{S_{t-1}}{N} - \kappa E_{t-1} \\
I_t &= I_{t-1} + \kappa E_{t-1} - \gamma I_{t-1} \\
R_t &= R_{t-1} + \gamma I_{t-1}
\end{aligned} \tag{A.1}$$

Here, E_t denotes the number of individuals that are exposed at day t, κ is the daily transition rate from exposed to infected, and $\epsilon \in [0, 1]$ measures the degree to which the exposed are less infectious than the infected. If $\epsilon = 0$, the exposed are not infectious at all, and we obtain the benchmark SEIR model. If $\epsilon = 1$, the exposed are as infectious as the infected, and the model is isomorphic to the standard SIR model.

We calibrate the parameters following Wang et al. (2020) who apply the benchmark SEIR model (with $\epsilon = 0$) to study the dynamics of COVID-19 in Wuhan. In particular, we use $\kappa = 1/5.2$ and $\gamma = 1/18$ as in Wang et al. (2020). Then, we set $\epsilon = 2/3$, following Ferguson et al. (2020) who assume that symptomatic individuals are 50% more infectious than the asymptomatic (that is, $\epsilon^{-1} = 1.5$). Finally, we choose β by targeting a basic reproduction number of $\mathcal{R}_0 = 2.6$, again as in Wang et al. (2020). In the model above, $\mathcal{R}_0$ is given by $\mathcal{R}_0 = \beta/\gamma + \beta\epsilon/\kappa$, implying $\beta = \mathcal{R}_0\gamma\kappa/(\gamma\epsilon + \kappa)$. The formula yields $\beta \approx 0.12$. Finally, we set $S_0 = 11 \times 10^6$ (approximating the population size of Wuhan), $E_0 = R_0 = 0$, and $I_0 = 1$.

The Monte Carlo design is as follows. First, we simulate the deterministic system in Eq. (A.1) using the parameters above. Then, we calculate the growth rate in the true number of infected individuals, i.e., $\text{gr}(I_t) = I_t/I_{t-1} - 1$. However, instead of knowing the true growth rate, the statistician is assumed to observe a noisy version of it given by

Figure A.1
Monte Carlo Simulation: Effects of Misspecification

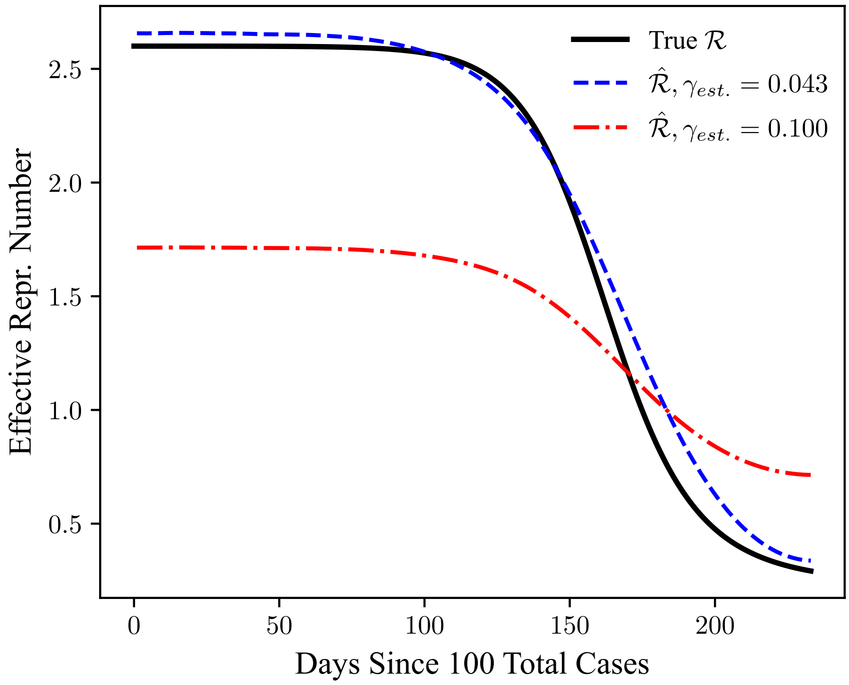

Notes: Estimates of the effective reproduction number ($\mathcal{R}_t$) when the true dynamics of the disease follow an SEIR model. We investigate two values for $\gamma_{\text{est.}}$, the transition rate from infected to recovered, that are used when estimating $\mathcal{R}_t$. First, we use the correct value of $\gamma_{\text{est.}} = (\gamma^{-1} + \kappa^{-1})^{-1} \approx 0.043$. Second, we use a misspecified values of $\gamma_{\text{est.}} = 1/10$. Average values from 10,000 Monte Carlo replications are shown. See text for more details.

$\widetilde{\text{gr}}(I_t) = \text{gr}(I_t) + \varepsilon_t$. Here, ε is an i.i.d. normal disturbance with mean zero and standard deviation of 0.10. The standard deviation of the disturbances is roughly equal to the range of the true growth rates. Hence, the amount of noise used in the simulation is fairly large. For each realization of the disturbances, we estimate $\mathcal{R}_t$ using our method. As in our empirical application, only data after 100 total cases have been reached is used.

We investigate two values for $\gamma_{\text{est.}}$ that are used when estimating $\mathcal{R}_t$. First, we consider a situation in which the statistician uses the correct time that individuals are infected, given by $\gamma_{\text{est.}} = (\gamma^{-1} + \kappa^{-1})^{-1}$ where γ and κ are the true parameter values of the SEIR model. Second, we investigate a case in which the statistician incorrectly thinks that individuals are infectious only for ten days ($\gamma_{\text{est.}} = 1/10$). We repeat the process for 10,000 Monte Carlo replications.

The results of the Monte Carlo simulation are shown in Figure A.1. When the statistician uses the correct number of days that an individual is infectious (that is, taking into

account the incubation time), the estimates of $\mathcal{R}_t$ from our method are very close to their true theoretical values. That is in spite of the fact that our estimator for $\mathcal{R}_t$ is derived assuming that the dynamics of the disease are described by an SIR model. However, we also show that if the statistician misspecifies the number of days than an individual is infectious (assuming 10 days instead of the true number of 23.2 days), the estimates of $\mathcal{R}_t$ are substantially biased, especially in the early stages of the epidemic. As is to be expected, underestimating the number of days that an individual is infectious leads to a downwards bias in the estimates of $\mathcal{R}_t$ early on in the epidemic (when $\mathcal{R}_t > 1$), and upwards bias when the true $\mathcal{R}_t$ falls below one. Overall, the results imply that the new method performs well when estimating $\mathcal{R}_t$ even when the true dynamics of the disease do not follow the SIR model, provided that the duration of infectiousness used in the estimation is sufficiently accurate.

A.6 Effects of Potential Data Issues

We now discuss the effects of various data issues on the performance of our estimator.

Reporting delays. In practice, data may be subject to significant reporting delays. For example, suppose that due to testing constraints there is a lag of ℓ days between the date that an individual becomes infected and the date on which the case is registered. In this case, the estimates of $\mathcal{R}_t$ would also be subject to delay of ℓ days. If there are significant reporting delays, one may first obtain, say, one-week-ahead forecasts of new cases, and then use these forecasts to construct a time series for I_t.

Imperfect detection. A natural worry with any estimator of $\mathcal{R}_t$ is that it may be substantially biased if not all of infected individuals are detected. Given the simplicity of our estimator, we can analytically assess the effects of imperfect detection.

Suppose that the true numbers of susceptible, infected, and recovered individuals are given by S_t^*, I_t^*, and $\mathcal{R}_t$, respectively. Their evolution is the same as in the SIR model in the main text in Eq. (1). However, we only observe $I_t = \alpha_t I_t^*$, where $\alpha_t \equiv I_t / I_t^*$ is the *detection rate*. In practice, α is typically less than one, although the mathematical calculation below does not require this.

With this notation, we have that

$$\text{gr}(I_t) = \text{gr}(\alpha_t)[1 + \text{gr}(I_t^*)] + \text{gr}(I_t^*) \approx \text{gr}(\alpha_t) + \text{gr}(I_t^*),$$

since $\text{gr}(\alpha_t) \times \text{gr}(I_t^*) \approx 0$ at a daily frequency; the approximation is exact in continuous time. Using the approximation above and Eq. (2) in the main text, we therefore obtain that the bias of the estimator under imperfect detection is given by

$$\hat{\mathcal{R}}_t - \mathcal{R}_t \approx \frac{1}{\gamma}\text{gr}(\alpha_t).$$

We now discuss several cases of practical importance:

- Constant detection rate ($\alpha_t = \alpha$). If the detection rate is constant over time, then our estimator is unbiased, and $\hat{\mathcal{R}}_t = \mathcal{R}_t$. Hence, for example, even if we only detect 10% of the infectives (but the fraction detected remains constant over time), the estimator remains unbiased. Note that if the number of tests increases over time, that is *not* inconsistent with $\alpha_t = \alpha$ given that the number of infected individuals is likely to be growing at the same time.
- Constant growth in the detection rate ($\text{gr}(\alpha_t) = g_\alpha$). If the growth rate of α_t is constant over time, then our estimate of $\mathcal{R}_t$ is biased upwards if $g_\alpha > 0$ and downwards if $g_\alpha < 0$. Note, however, that we are often mostly interested in

the *trend* of $\mathcal{R}_t$ over time and whether the trend is affected by various policy interventions. The trend in $\mathcal{R}_t$ is estimated accurately even if $g_\alpha \neq 0$. Intuitively, constant growth in the detection rate leads to a level bias, but the slope is still estimated correctly.

- Detection rate converges over time ($\alpha_t \to \alpha$). The final case of interest occurs when the detection rate converges to a constant over time. For example, if everyone is detected towards the end of the epidemic, we would have $\alpha_t \to 1$. Since our method uses Kalman-filtering techniques to estimate the growth rate of I_t, transient fluctuations in α_t would have a limited effect on the estimates of $\mathcal{R}_t$ later on in the sample. Given that we are often precisely interested in the behavior of $\mathcal{R}_t$ in the later stages of the epidemic (when the detection rate is likely fairly constant), our method would still yield reliable estimates.

To provide a quantitative illustration, we perform a small-scale Monte Carlo study. We choose the parameters of the simulation to match our empirical estimates. First, we use our empirical estimates of $\mathcal{R}$ for the world as a whole for the first 50 days of the sample as the true values of $\mathcal{R}$. From these values of $\mathcal{R}$, we calculate the true (but unobserved) values of the growth rate of the number of infected individuals as $\mu_t \equiv \text{gr}(I_t^*) = \gamma(\mathcal{R}_t - 1)$, using $\gamma = 1/7$. Finally, the observed growth rate—as seen by the statistician—is generated as $\text{gr}(I_t) = \text{gr}(\alpha_t)(1 + \mu_t) + \mu_t + \varepsilon_t$. We use the empirical estimate of σ_ε^2 to simulate ε_t shocks (i.i.d. draws from a normal distribution with mean zero). We then apply our estimator to the generated data on $\text{gr}(I_t)$, using the same value of $\gamma = 1/7$ to back out $\mathcal{R}$.

In the simulation we consider three underdetection scenarios:

- *Constant underdetection*. Given our analytical results, it is immaterial what percentage of cases is detected, as long as that percentage is constant over time.
- *Ramp-up in testing*. Next, we consider a situation in which—due to an increase in the number of tests performed—the fraction of detected infected individuals goes up from $\alpha_0 = 0.10$ to $\alpha_{14} = 0.15$ (an increase of 50%) over a period of two weeks. After the two weeks, the detection probability remains constant at 0.15. Again, the precise values of α_0 and α_{14} are irrelevant, and what matters is the growth rate in the detection rate.
- *Stochastic underdetection*. Finally, we suppose that the detection rate satisfies $\text{gr}(\alpha_t) = \phi\,\text{gr}(\alpha_{t-1}) + \nu_t$ where ν_t is an i.i.d. normally distributed shock. Intuitively, the detection rate is assumed to be unconditionally constant, but its growth rate is stochastic and follows an AR(1) process. We set the persistence parameter to $\phi = 0.75$ in order to allow for fairly long-lasting deviations from the average

Figure A.2

Monte Carlo Simulation: Effects of Underdetection

(a) Average Estimates

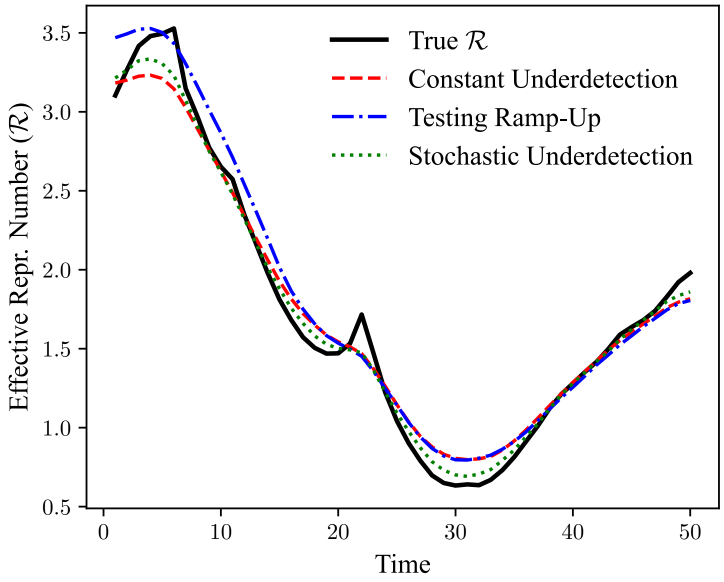

(b) Estimation Accuracy

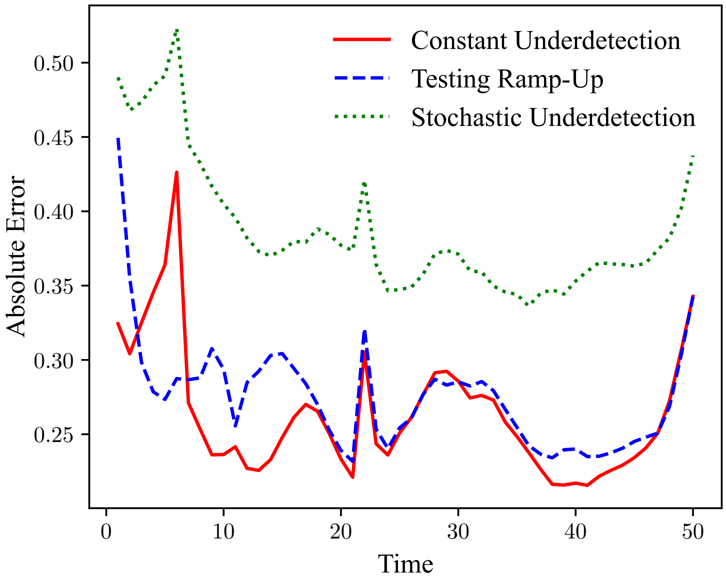

Notes: Estimates of the effective reproduction number ($\mathcal{R}_t$) when the true number of infected individuals is measured with error. The true values for $\mathcal{R}_t$ are given by our empirical estimates of $\mathcal{R}_t$ for the world as a whole in the first 50 days of the sample, and the standard deviation of the irregular component is chosen to match our empirical estimates. *Constant Underdetection*: A constant fraction of infected individuals is detected. *Testing Ramp-Up*: The fraction of detected individuals increases by 50% in the first two weeks of the sample. *Stochastic Underdetection*: The fraction of detected individuals is stochastic, and its growth rate follows an AR(1) process. Average values from 1,000 Monte Carlo replications are shown. *Top panel*: Average estimates. *Bottom panel*: average absolute error of the estimates.

Figure A.3

Monte Carlo Simulation: Coverage Frequency

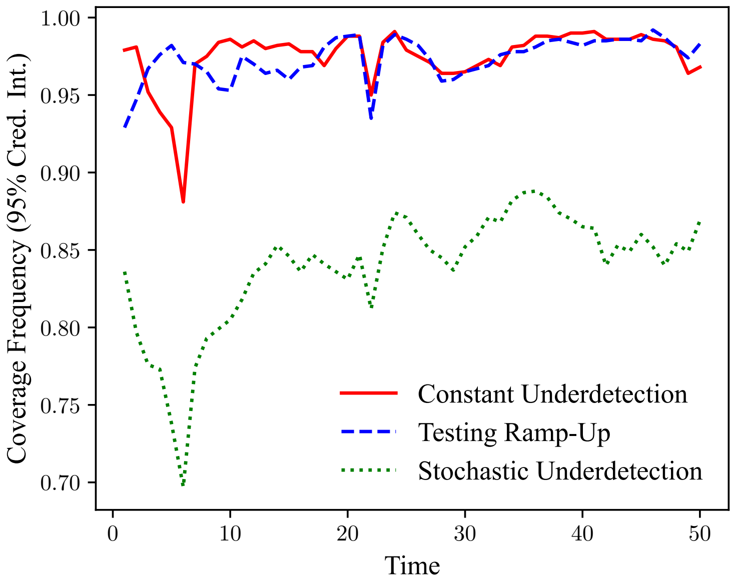

Notes: Coverage frequency of 95% credible intervals: Monte Carlo results. We investigate three scenarios for measurement error. *Constant Underdetection*: A constant fraction of infected individuals is detected. *Testing Ramp-Up*: The fraction of detected individuals increases by 50% in the first two weeks of the sample. *Stochastic Underdetection*: The fraction of detected individuals is stochastic, and its growth rate follows an AR(1) process. Average values from 1,000 Monte Carlo replications are shown. See text and Figure A.2 for more details.

detection rate. To ensure that the variance of gr(I_t) remains constant across simulations (to ensure an apples-to-apples comparison), we suppose that 50% of the noise in the observed growth rate comes from variation in α_t, with the other 50% coming from the ε_t shocks. (Here, by "noise" we refer to the variation in gr(I_t) that is not solely due to the variation in μ_t, namely, gr$(\alpha_t)(1 + \mu_t) + \varepsilon_t$.) Denoting our estimates of the variance of the growth rate by $\hat{\sigma}_\mu^2$ and the variance of the irregular component by $\hat{\sigma}_\varepsilon^2$, we therefore set $\mathbb{V}\text{ar}[\nu_t] = (1 - \phi^2)\hat{\sigma}_\varepsilon^2/[2(1 + \hat{\sigma}_\mu^2)]$ and $\mathbb{V}\text{ar}[\varepsilon_t] = \hat{\sigma}_\varepsilon^2/2$. We draw the initial value for gr(α_0) from its unconditional distribution.

The results of the Monte Carlo simulation are summarized in Figure A.2. As seen in the top panel, the estimated average effective reproduction numbers are fairly close to their theoretical values in all three scenarios. In the *Testing Ramp-Up* scenario, the estimates are biased upwards at the beginning of the sample, but the amount of bias is quantitatively relatively small. In addition, the estimates converge to those in the other two scenarios quite quickly. In all three scenarios, the estimates are able to pick up

the reversal in the trend of $\mathcal{R}$ that happens at around time 30. The bottom panel of the figure plots the average absolute error of the estimates. As expected, the estimates are least accurate in the *Stochastic Undertesting* case, and mostly within 0.25–0.30 of the true $\mathcal{R}$ in the *Constant Underdetection* and *Testing Ramp-Up* scenarios.

Finally, in Figure A.3 we provide Monte Carlo estimates of the coverage frequency of credible intervals obtained by our estimation procedure. As seen in the graph, the credible intervals in the *Constant Undertesting* and *Testing Ramp-Up* scenarios have good coverage properties, with conservative confidence bounds. The credible bounds are narrower in the *Stochastic Underdetection* scenario, resulting in lower than nominal coverage frequency. However, the size distortion appears not too severe, especially considering the small sample size.

Imported cases. Our estimates may be biased if the fraction of cases that is imported changes over time (the previous results on imperfect detection apply to misclassification because of imported cases, too). If the source of infections is known, it is possible to correct for the issue by simply not including imported cases when constructing the time series for I_t.

A.7 Estimation Details

To estimate $\mathcal{R}_t$ of COVID-19, we use Bayesian filtering methods. We employ the following strategy to calibrate the prior distributions. First, we estimate a local-level model for gr(I_t) using a frequentist Kalman filter with diffuse initial conditions. In particular, we estimate the following model:

$$\begin{aligned} \text{gr}(I_t) &= \mu_t + \varepsilon_t, \quad && \varepsilon_t \sim \text{i.i.d. } \mathcal{N}(0, \sigma_\varepsilon^2) \\ \mu_t &= \mu_{t-1} + \eta_t, && \eta_t \sim \text{i.i.d. } \mathcal{N}(0, \sigma_\eta^2) \end{aligned} \tag{A.2}$$

The model is the same as Eq. (5) in the main text, except for a slight simplification in notation.

The procedure yields maximum likelihood estimates of σ_ε^2 (variance of the irregular component) and the signal-to-noise ratio $q \equiv \sigma_\eta^2/\sigma_\varepsilon^2$ for each country in the sample (with σ_η^2 denoting the variance of the level component). We then use the distribution of $\hat{\sigma}_\varepsilon^2$ and $\hat{q}$ across countries to calibrate the priors for the precision of the irregular component ($1/\sigma_\varepsilon^2$) and the signal-to-noise ratio (q). To ensure that the priors are not too "dogmatic," we inflate the variance of the estimates by a factor of 3 when calibrating the prior distributions. We use a gamma prior for both the signal-to-noise ratio and the precision of the irregular component, and we calibrate the parameters of the gamma distribution by matching the expected value and variance of the gamma-distributed random variables to their sample counterparts. Finally, we use a fairly uninformative normal prior for the initial value of the smoothed growth rate. The resulting priors are given in Table A.1.

Intuitively, these priors shrink the estimates of the precision and signal-to-noise ratio for each country towards their grand mean (average across countries). Such Bayesian shrinkage ensures that the parameter estimates are well behaved even though the sample

Table A.1

Priors

Parameter	Prior
Precision of irregular component ($1/\sigma_\varepsilon^2$)	Gamma$(0.17985, 0.00099)$
Signal-to-noise ratio ($q \equiv \sigma_\eta^2/\sigma_\varepsilon^2$)	Gamma$(0.07781, 0.54261)$
Initial value (μ_0)	$\mathcal{N}(0.35, 0.5^2)$

Notes: Priors used in the Bayesian estimation of $\mathcal{R}_t$. See text for description on how the priors for the precision of the irregular component ($1/\sigma_\varepsilon^2$) and the signal-to-noise ratio ($q \equiv \sigma_\eta^2/\sigma_\varepsilon^2$) are calibrated based on cross-country frequentist estimates.

size for many countries is fairly small, and the data are often noisy. We use the Stan programming language (Gelman, Lee, and Guo, 2015) to specify and estimate the Bayesian model. In particular, we use the `pystan` interface to call Stan from Python.

A.8 Empirical Validation

In this section, we perform two empirical validation exercises to check the performance of our estimates in practice.

Since our estimates are based on data on new cases, they may be misleading if new cases are subject to significant measurement problems. To help assuage this concern, we now perform the following exercise. We ask whether *current* values of $\mathcal{R}_t$ help predict *future* growth in deaths. Since deaths are likely to be measured more accurately, this exercise provides a test of whether our estimates contain meaningful information and are not contaminated by data problems.

Formally, we consider the following regression:

$$\text{gr}(d_{i,t+1}) = \alpha_i + \beta(\hat{\mathcal{R}}_{i,t} - 1) + u_{i,t},$$

Figure A.4

$\mathcal{R}_t$ and Future Deaths

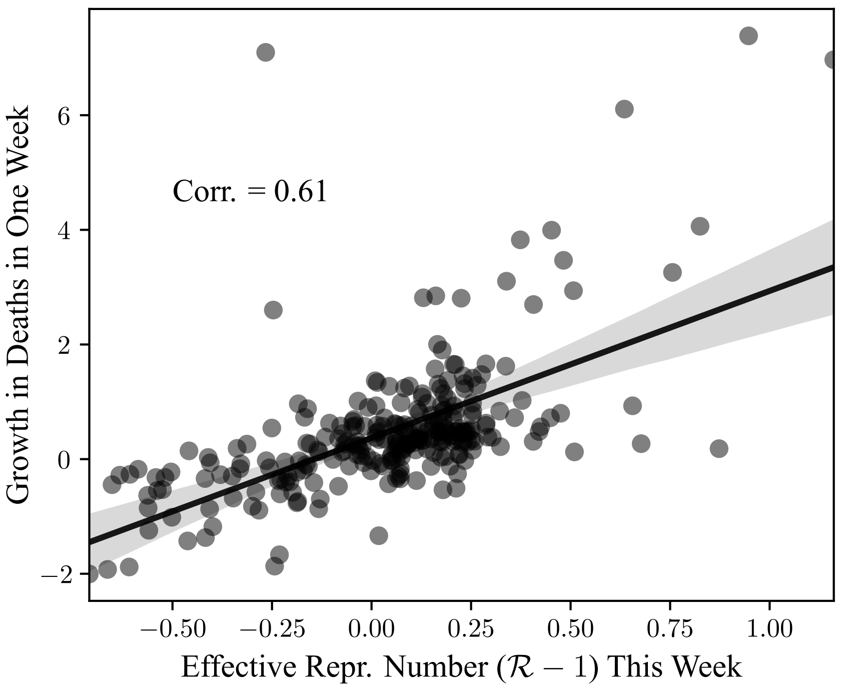

Notes: Relationship between current estimates of the effective reproduction number ($\mathcal{R}_t$) and the growth rate of the number of new deaths in one week. The data is aggregated to a weekly frequency. Both variables are residualized to subtract country fixed effects by performing the within transformation. Only data after the cumulative number of deaths reaches 50 is included in the scatter plot. We include all countries in the John Hopkins database for which we have at least 20 observations after the outbreak. We remove data for the week of 2020-04-13–2020-04-19 in China that contain a large number of deaths that were previously unrecognized.

Figure A.5

$\mathcal{R}_t$ and Deaths in Two Weeks

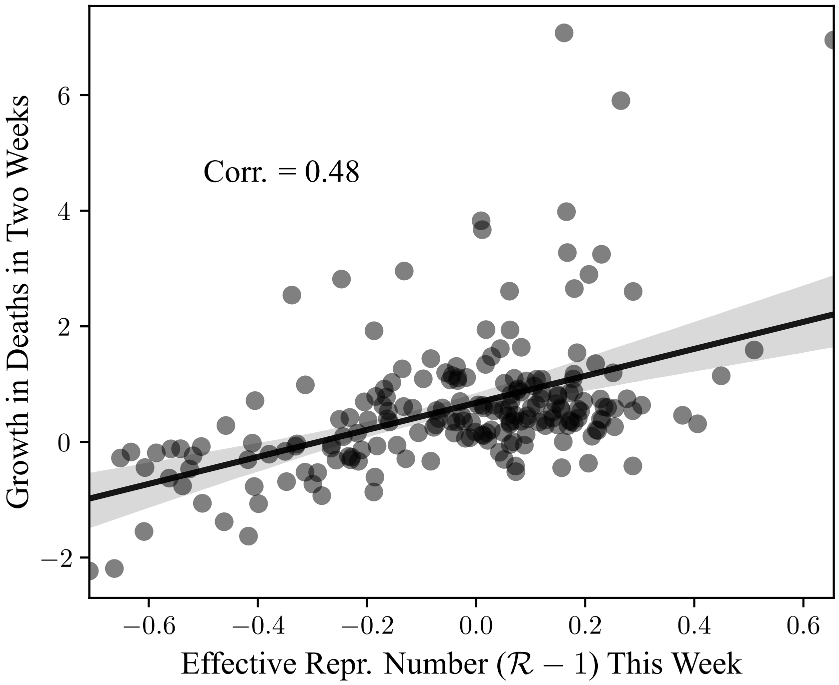

Notes: Relationship between current estimates of the effective reproduction number ($\mathcal{R}_t$) and the growth rate of the number of new deaths in two weeks. The data is aggregated to a weekly frequency. Both variables are residualized to subtract country fixed effects by performing the within transformation. See Figure A.4 for more details.

where i denotes a particular country, and t indexes calendar weeks. Although our original data is daily, we aggregate to a weekly frequency; otherwise, measures of the growth rate of new deaths are too noisy. In addition, we only include weeks after the cumulative number of COVID-19 deaths has reached 50. After imposing these sample restrictions, we are left with 270 country-week observations across 68 countries. Given that we have panel data, we can include country fixed effects α_i to account for time-invariant unobserved heterogeneity (such as differences in average age—a key correlate of COVID-19 mortality (Verity et al., 2020)—or family structures). The relationship given above is predicted by the baseline SIR model. Letting CFR $= d_t/I_{t-\ell}$ denote the case fatality rate (assumed to be constant over time), with ℓ standing for the average time between becoming infected and death, we have that $\text{gr}(d_t) = \text{gr}(I_{t-\ell})$, yielding the regression equation above.

The relationship is shown in Figure A.4. In the scatter plot, both variables are residualized to remove country fixed effects. We observe a strong positive relationship be-

Figure A.6

$\mathcal{R}_t$ and Past Mobility

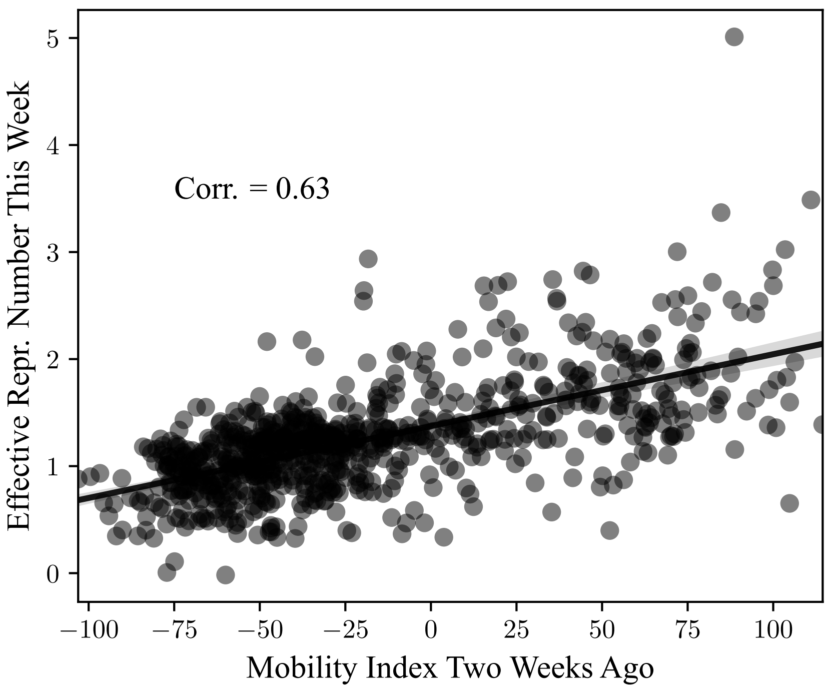

Notes: Relationship between current estimates of the effective reproduction number ($\mathcal{R}_t$) and value of the movement index two weeks ago (first principal component of the six movement categories in Google (2020)). The data is aggregated to a weekly frequency. Both variables are residualized to subtract country fixed effects by performing the within transformation. We include all countries in the John Hopkins database for which we have at least 20 observations after the outbreak.

tween the value of $\mathcal{R}_t$ this week and the growth in deaths one week later (corr. = 0.61). In Figure A.5, we demonstrate that there is also positive correlation (corr. = 0.48) between $\mathcal{R}_t$ and deaths two weeks later. We note that while the average medical duration from the onset of symptoms to death for COVID-19 is longer than two weeks (around 18 days, see Verity et al., 2020), the duration from *reported* cases to deaths is likely to substantially shorter because of reporting delays. For example, Hortaçsu, Liu, and Schwieg (2020) assume that new cases of COVID-19 are reported with a lag of 8 days in their baseline calculations (5 days for symptoms to appear, consistent with the evidence from Lauer et al. (2020) and Park et al. (2020), as people are unlikely to be tested without exhibiting symptoms, and an additional 3 days to capture delays in obtaining test results, based on andecdotal reports from the US). Since deaths are likely reported in a timely manner, if new cases are reported with a lag of 8 days, we would expect an average duration of around 10 days ($\approx$1.43 weeks) between reported cases and reported deaths.

As a second validation check, we ask whether our estimates of $\mathcal{R}_t$ are correlated with past movement data, as it should be if the estimates are meaningful. For information on movement, we use aggregated smartphone location data collected by Google and published in their "COVID-19 Community Mobility Reports" (Google, 2020). Google provides data on percentage changes in movement for six types of places: (i) groceries and pharmacies; (ii) parks; (iii) transit stations; (iv) retail and recreation; (v) residential; and (vi) workplaces. Since the six categories are strongly correlated, we take the first principal component of the six categories (the first principal component explains 83.03% of the total variance in the data). We refer to the first principal component as the "Mobility Index." As before, we only consider weeks after the cumulative number of confirmed COVID-19 cases in the country reaches 100. After imposing these restrictions, we are left with 792 country-week observations over 100 countries. Note that the number of countries is less than 124 because Google does not provide mobility data for all countries in our original sample. As shown in Figure A.6, current estimates of $\mathcal{R}_t$ are strongly correlated with the value of the mobility index two weeks ago (corr. = 0.63).

For both validation exercises performed in the present section, we include all countries for which we have at least 20 observations after the onset of the epidemic (100 cumulative cases of COVID-19 reached) and satisfy any additional sample restrictions, as outlined above. If we narrow the sample down to countries with more and higher-quality data—such as the sample of European countries analyzed in the main text—the correlations generally become substantially stronger. Hence, we consider the tests of the present section to be conservative.

A.9 Plot of Raw Growth Rates

Figure A.7

Growth Rate of the Number of Infected Individuals

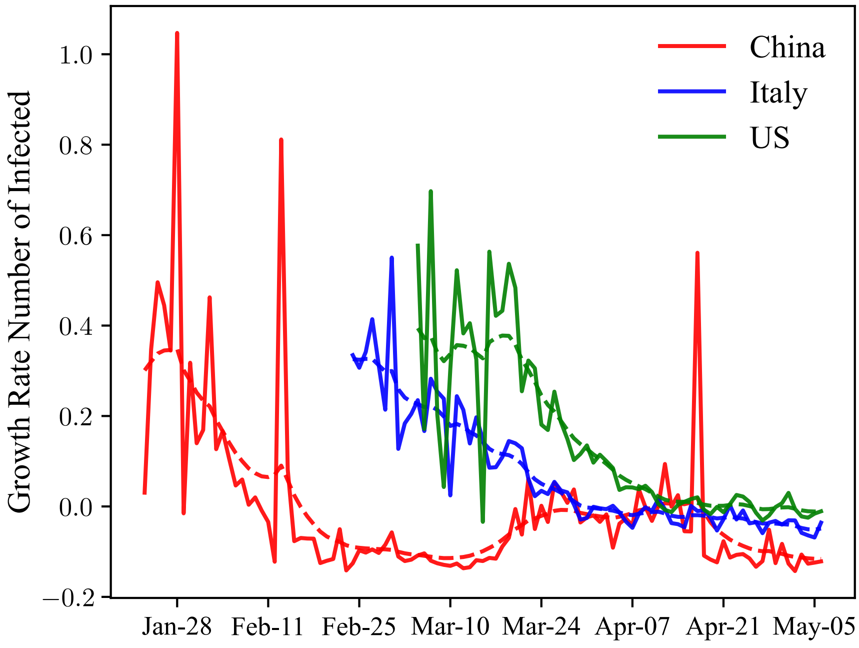

Notes: Raw data for the growth rate of the number of infected individuals (solid lines) and our estimate of its time-varying average (dashed lines) for China, Italy, and the US.

A.10 Kalman Filter vs Smoother Estimates

Figure A.8

Filtered and Smoothed Estimates: China

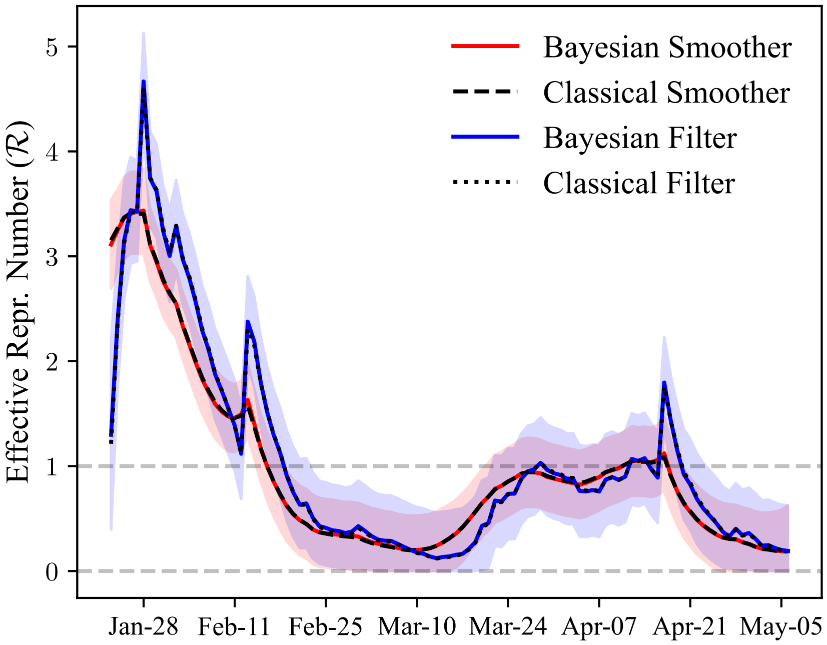

Notes: Estimated effective reproduction number ($\mathcal{R}$) for China: filtered and smoothed estimates, using both Bayesian and classical estimation procedures. The Bayesian estimates are given by our baseline estimation procedure, as explained in the text. The classical estimates are obtained by maximum likelihood estimation (with diffuse initial conditions). The smoothed estimates use information from the full sample, while the filtered estimates at time t only use information up to time t. 65% credible bounds shown by the shaded areas.

A.11 Power Analysis

In this section, we study the statistical power of the event-study analysis in the main text using a Monte Carlo simulation.

We now describe the design of the power study. Intuitively, we simulate data using a stochastic process that is calibrated to match the properties of the observed data. We then simulate a sharp drop in the effective reproduction number—say, because of a lockdown. We apply our estimator to the simulated data and ask how often this abrupt change is detected by the estimation procedure.

To simplify the notation, we use the parametrization in Eq. (A.2). Optimal nowcasts from the local-level model in the steady state can be written as (Muth, 1960; Shephard, 2015, Section 3.4):

$$m_t = \omega y_t + (1-\omega)m_{t-1}, \omega = \frac{q+\sqrt{q^2+4q}}{2+q+\sqrt{q^2+4q}}, \tag{A.3}$$

where we denote $y_t \equiv \text{gr}(I_t)$, $\mu_t \equiv \gamma(\mathcal{R}_t - 1)$, $m_t \equiv \mathbb{E}_t[\mu_t]$, $q \equiv \sigma_\eta^2/\sigma_\varepsilon^2$ is the signal-to-noise ratio, and ω is the steady-state Kalman gain. Hence, nowcast errors, $m_t - \mu_t$, follow an AR(1) process with

$$m_t - \mu_t = (1-\omega)(m_{t-1} - \mu_{t-1}) + \{\omega\varepsilon_t - (1-\omega)\eta_t\}.$$

Given that the shocks ε_t and η_t are uncorrelated, the variance of nowcast errors is

$$\mathbb{V}\text{ar}(m_t - \mu_t) = \frac{\omega^2\sigma_\varepsilon^2 + (1-\omega)^2 q\sigma_\varepsilon^2}{1-(1-\omega)^2}. \tag{A.4}$$

The design of the power analysis is as follows:

1. We set $\gamma = 1/7$, and calibrate the remaining parameters of the data-generating process (q and σ_ε^2) using the median values of the empirical estimates in the main text. The resulting parameter values are given in Table A.2.
2. We simulate $\mu_t = \gamma(\mathcal{R}_t - 1)$. We initially set $\mu_0 = 1/7$, implying an effective reproduction number of 2. At time 1, we simulate an abrupt decline in $\mathcal{R}_t$ by setting $\mu_1 = 1/14$, yielding a new effective reproduction number of 1.5, or a decline of 25%. For $2 \leq t \leq 14$, we simulate μ_t as a random walk, as in Eq. (A.2).
3. We simulate the observed growth rate of the number of infected individuals, $y_t = \text{gr}(I_t)$, as $y_t = \mu_t + \varepsilon_t$ where ε_t is an i.i.d. normal random variable with mean zero and variance σ_ε^2.
4. We simulate the nowcasts m_t. We draw the initial nowcast m_0 from a normal

Table A.2

Power Analysis: Parameter Values

γ	q	σ_ε^2	ω
0.143	0.073	0.015	0.236

Notes: Parameter values used in the power analysis. The parameters values for q (signal-to-noise ratio) and σ_ε^2 (variance of the irregular component) are given by the median estimates from the 14 European countries considered in the empirical analysis in the main text. The mean duration of infectiousness is assumed to be $\gamma^{-1} = 7$, and the Kalman gain ω is calculated from Eq. (A.3).

distribution with mean $1/7$ and variance given in Eq. (A.4), and simulate further values of m_t by the recursion in Eq. (A.3). The estimated value of $\mathcal{R}_t$ is then given by our estimator.

5. We repeat steps 2–4 for 14 times, to simulate data for 14 "countries", as in the empirical application, and obtain estimates of the effective reproduction number by averaging across the 14 "countries".
6. We repeat steps 2–5 for 10,000 Monte Carlo replications.

The results of the power analysis are shown in Figure A.9. We observe that in 95% of the simulations, the change in $\mathcal{R}_t$ is detected as soon as two days after the drop in $\mathcal{R}_t$. Hence, the analysis in the main text appears sufficiently powerful to detect moderate changes in $\mathcal{R}_t$. The key reason why the analysis has high statistical power, even though the signal-to-noise ratio is quite low (see Table A.2) is that data from multiple countries are used to obtain cross-country averages. This feature of the estimation procedure reduces estimation error substantially. While the signal-to-noise ratio is fairly low, we also note that the weight placed on data is that are more than one-week old is only $(1-\omega)^7 \approx 15.2\%$. Hence, one week after the change in $\mathcal{R}_t$, the estimates of $\mathcal{R}_t$ are based primarily on data received after the change in $\mathcal{R}_t$.

The power analysis in the current section is arguably conservative. Specifically, we assume that after the abrupt decline, $\mathcal{R}_t$ follows a random walk rather than staying fixed at the new level. As a result, as time goes on, the estimates of $\mathcal{R}_t$ become more "spread out" across simulations, as is visible towards the end of Figure A.9.

Figure A.9

Power Analysis: Monte Carlo Results

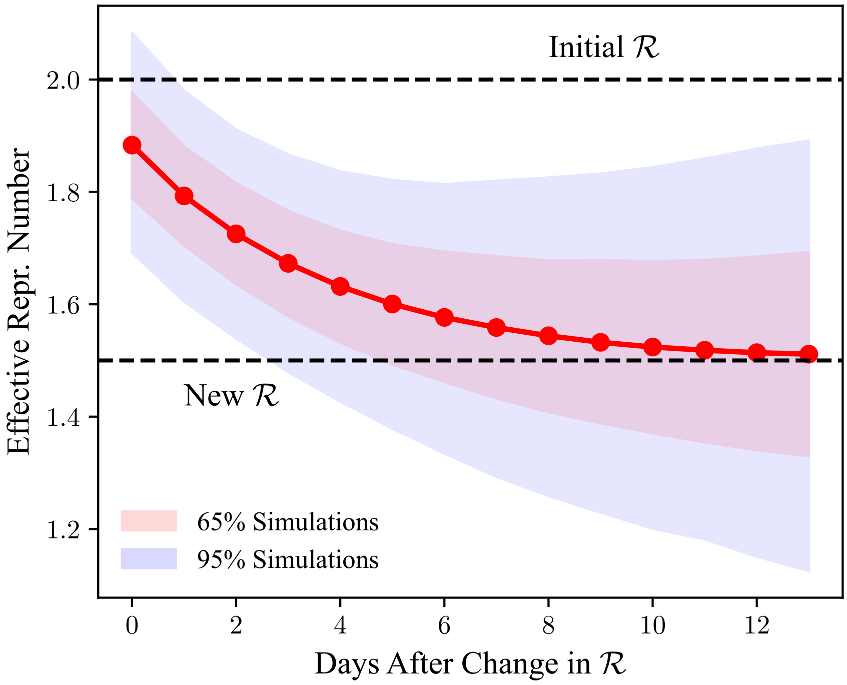

Notes: Power study of the statistical analysis in the main text (effects of non-pharmaceutical interventions on $\mathcal{R}_t$, the effective reproduction number). We simulate an abrupt change in $\mathcal{R}_t$ from 2.0 to 1.5 using a data-generating process that is calibrated to match our empirical estimates in the main text. We then apply our estimator to the simulated data and ask how often the change is detected by the estimation procedure. The solid line gives the average estimate of $\mathcal{R}_t$, while the shaded lines denote 65% and and 95% of simulations (in particular, the shaded area for 65% of simulations is given by the 17.5 and 82.5 percentiles of the estimated $\mathcal{R}_t$ across simulations, and the shaded area for 95% of simulations is given by the 2.5 and 97.5 percentiles of the estimated $\mathcal{R}_t$'s). 10,000 Monte Carlo replications used.

A.12 Comparison With EpiEstim Estimates

In this section, we compare our estimates of $\mathcal{R}$ for COVID-19 with estimates obtained using the method of Cori et al. (2013); see also Thompson et al. (2019). The method proposed by Cori et al. (2013)—arguably the most widely used approach to estimating the effective reproduction number of an infectious disease—is implemented in a popular R package `EpiEstim`.

First, we download estimates of $\mathcal{R}$ obtained using `EpiEstim` provided by Xihong Lin's Group in the Department of Biostatistics at the Harvard Chan School of Public Health (http://metrics.covid19-analysis.org/). These estimates, just as in our case, are obtained using data from the John Hopkins CSSE repository. The parametrization used in the `EpiEstim` estimation assumes a time window of 7 days, and a gamma-distributed serial interval with a mean of 5.2 days and a standard deviation of 5.1 days. Next, we merge these estimates to our full sample of estimates. We restrict attention to countries for which we have at least 20 contemporaneous estimates from each of the two different methods. That leaves us with 108 countries and, on average, 42.14 time-series observations per country. Finally, we calculate the Pearson correlation coefficient between the two estimates of $\mathcal{R}$ for each country.

The results of this exercise are given in Table A.3. The two sets of estimates are highly correlated, with the average correlation coefficient equal to 0.80 (median: 0.89). The interquartile range is 0.78–0.95. These findings suggest that the two estimates are highly consistent with each other, despite very different estimation methods and underlying assumptions.

Table A.3

Comparison With Cori et al Estimates

No. Countries	Avg. Sample Size	Avg. Corr.	P5	P25	P50	P75	P95
108	42.14	0.80	0.14	0.78	0.89	0.95	0.99

Notes: Correlation of our estimates with estimates of the effective reproduction number obtained using the method of Cori et al. (2013), as implemented in the `EpiEstim` R package. For each country in the sample for which we have at least 20 contemporaneous estimates, we calculate the Pearson correlation coefficient between the two sets of estimates. *No. Countries*: Number of countries in the sample. *Avg. Sample Size*: Average number of time-series observations per country. *Avg. Corr.*: Average correlation coefficient across countries. *P5* to *P95*: Percentiles of the correlation coefficients across countries.

A.13 Additional Event-Study Graphs

Figure A.10

$\mathcal{R}_t$ and Policy Interventions: Bans of Public Events

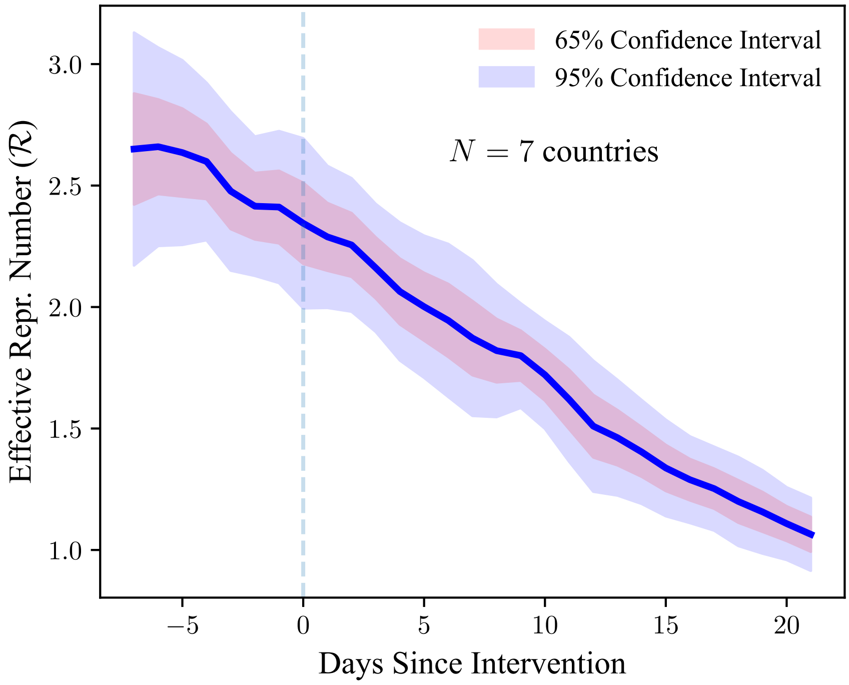

Notes: The graph plots the estimated effective reproduction number ($\mathcal{R}_t$) one week before and three weeks after public events are banned in a country. The original sample consists of 14 European countries studied by Flaxman et al. (2020). For the event-study graph, we restrict the sample to countries for which data on $\mathcal{R}_t$ is available for the whole event window. Heteroskedasticity-robust confidence bounds are shown by the shaded areas.

Figure A.11

$\mathcal{R}_t$ and Policy Interventions: Case-Based Measures

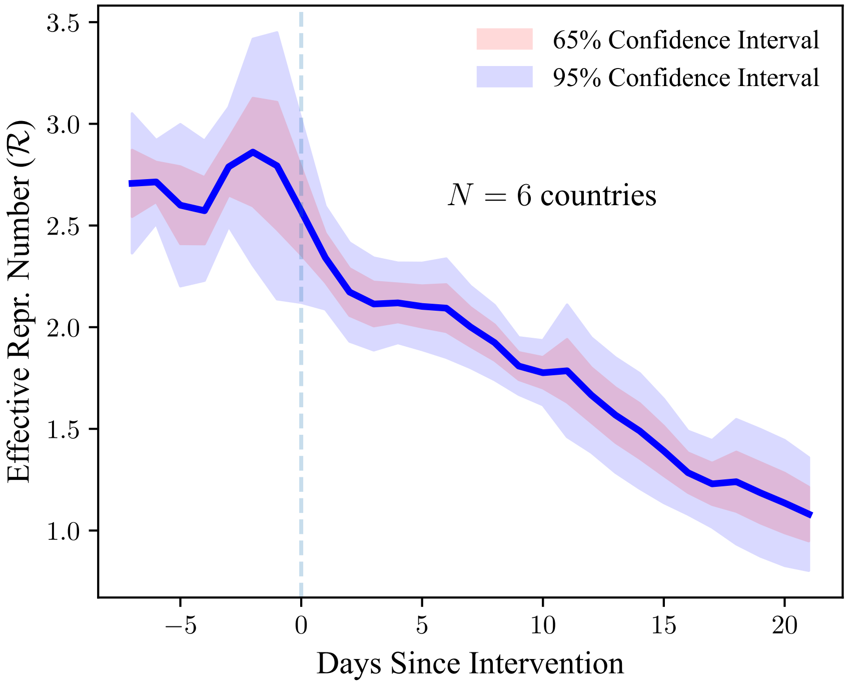

Notes: The graph plots the estimated effective reproduction number ($\mathcal{R}_t$) one week before and three weeks after case-based measures are introduced in a country. The original sample consists of 14 European countries studied by Flaxman et al. (2020). For the event-study graph, we restrict the sample to countries for which data on $\mathcal{R}_t$ is available for the whole event window. Heteroskedasticity-robust confidence bounds are shown by the shaded areas.

Figure A.12

$\mathcal{R}_t$ and Policy Interventions: School Closures

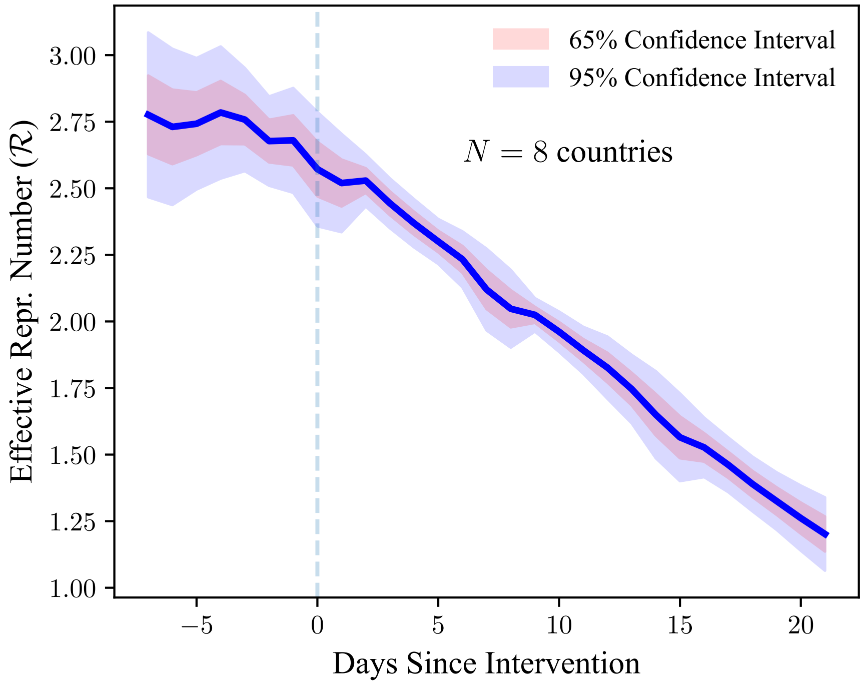

Notes: The graph plots the estimated effective reproduction number ($\mathcal{R}_t$) one week before and three weeks after school closures are ordered in a country. The original sample consists of 14 European countries studied by Flaxman et al. (2020). For the event-study graph, we restrict the sample to countries for which data on $\mathcal{R}_t$ is available for the whole event window. Heteroskedasticity-robust confidence bounds are shown by the shaded areas.

Figure A.13

$\mathcal{R}_t$ and Policy Interventions: Social Distancing

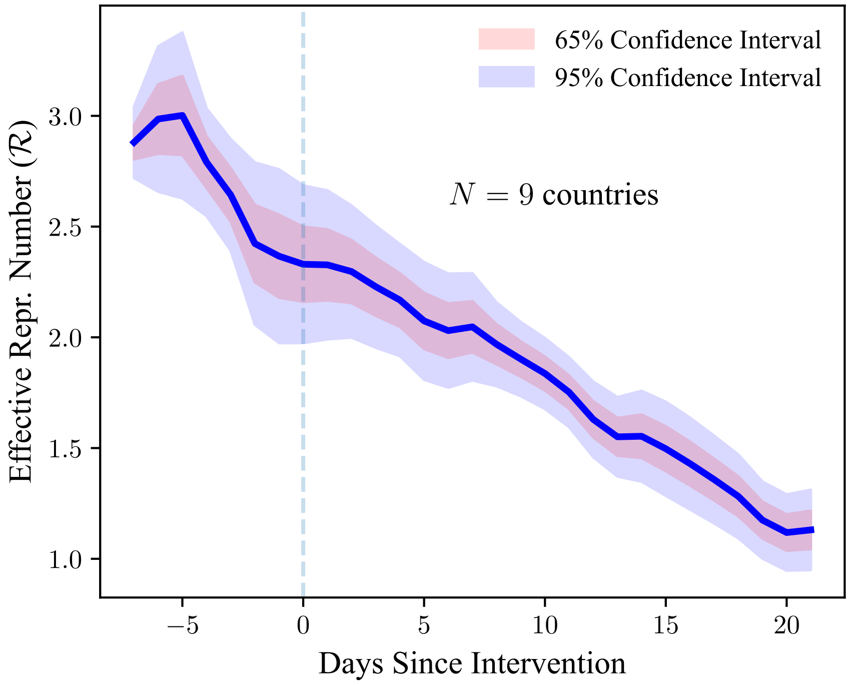

Notes: The graph plots the estimated effective reproduction number ($\mathcal{R}_t$) one week before and three weeks after social distancing is encouraged in a country. The original sample consists of 14 European countries studied by Flaxman et al. (2020). For the event-study graph, we restrict the sample to countries for which data on $\mathcal{R}_t$ is available for the whole event window. Heteroskedasticity-robust confidence bounds are shown by the shaded areas.

A.14 Regressions with NPIs Included Separately

Table A.4

Effective Reproduction Number and NPIs

	Dependent Variable: $\log(R_t)$				
	(1)	(2)	(3)	(4)	(5)
Lockdown	-0.14***				
	(0.04)				
Public Events		0.07			
		(0.04)			
School Closure			-0.17***		
			(0.05)		
Self Isolation				-0.15***	
				(0.05)	
Social Distancing					-0.13**
					(0.06)
N	855	855	855	855	855
R^2	0.86	0.86	0.86	0.86	0.86
Country FE	✓	✓	✓	✓	✓
Days-Since-Outbreak FE	✓	✓	✓	✓	✓
Mobility Controls					
Testing Controls					

$^{*}p<0.1$; $^{**}p<0.05$; $^{***}p<0.01$

Notes: Results of panel-data regressions of the (log of) effective reproduction number ($\mathcal{R}_t$) on indicator variables that are equal to 1 after the introduction of a non-pharmaceutical intervention (NPI) and 0 before the introduction. The regressions are similar to those in Table 2 in the main text except that the intervention variables are included separately (one-at-a-time). See the main text for more details.

A.15 GATHER Checklist

Item #	Checklist item	Reported on page #
**Objectives and funding**		
**1**	Define the indicator(s), populations (including age, sex, and geographic entities), and time period(s) for which estimates were made.	3, 5
**2**	List the funding sources for the work.	13
**Data inputs**		
For all data inputs from multiple sources that are synthesized as part of the study:		
**3**	Describe how the data were identified and how the data were accessed.	3, 5
**4**	Specify the inclusion and exclusion criteria. Identify all ad-hoc exclusions.	3, 5
**5**	Provide information on all included data sources and their main characteristics. For each data source used, report reference information or contact name/institution, population represented, data collection method, year(s) of data collection, sex and age range, diagnostic criteria or measurement method, and sample size, as relevant.	3, 8
**6**	Identify and describe any categories of input data that have potentially important biases (e.g., based on characteristics listed in item 5).	A8, A9
For data inputs that contribute to the analysis but were not synthesized as part of the study:		
**7**	Describe and give sources for any other data inputs.	3, 8

Item #	Checklist item	Reported on page #
For all data inputs:		
**8**	Provide all data inputs in a file format from which data can be efficiently extracted (e.g., a spreadsheet rather than a PDF), including all relevant metadata listed in item 5. For any data inputs that cannot be shared because of ethical or legal reasons, such as thirdparty ownership, provide a contact name or the name of the institution that retains the right to the data.	1, 2
**Data analysis**		
**9**	Provide a conceptual overview of the data analysis method. A diagram may be helpful.	3, 4, A13
**10**	Provide a detailed description of all steps of the analysis, including mathematical formulae. This description should cover, as relevant, data cleaning, data preprocessing, data adjustments and weighting of data sources, and mathematical or statistical model(s).	3, 4, A13
**11**	Describe how candidate models were evaluated and how the final model(s) were selected.	3, 4, A5, A6, A8, A9, A13,
**12**	Provide the results of an evaluation of model performance, if done, as well as the results of any relevant sensitivity analysis.	A5, A6, A8, A9, A10, A11 A13, A15, A16, A17, A18, A24
**13**	Describe methods for calculating uncertainty of the estimates. State which sources of uncertainty were, and were not, accounted for in the uncertainty analysis.	4, A13
**14**	State how analytic or statistical source code used to generate estimates can be accessed.	2

Item #	Checklist item	Reported on page #
**Results and discussion**		
**15**	Provide published estimates in a file format from which data can be efficiently extracted.	2
**16**	Report a quantitative measure of the uncertainty of the estimates (e.g. uncertainty intervals).	6, 8, 10
**17**	Interpret results in light of existing evidence. If updating a previous set of estimates, describe the reasons for changes in estimates.	6, 7, 9
**18**	Discuss limitations of the estimates. Include a discussion of any modelling assumptions or data limitations that affect interpretation of the estimates.	11, 12

Notes: GATHER checklist (gather-statement.org) to facilitate the evaluation and replication of our empirical analysis. References to numbers in the Supplementary Appendix are prefixed with an "A." Hence, for example, "A15" refers to page 15 in the Supplementary Appendix, while "15" refers to page 15 in the main text.

References

Carter, C. K., Kohn, R., 1994. On Gibbs sampling for state space models. Biometrika 81 (3), 541–553.

Chowell, G., Brauer, F., 2009. The basic reproduction number of infectious diseases: Computation and estimation using compartmental epidemic models. In: Chowell, G., Hyman, J. M., Bettencourt, L. M. A., Castillo-Chavez, C. (Eds.), Mathematical and Statistical Estimation Approaches in Epidemiology. Springer, Ch. 1, pp. 1–30.

Cori, A., Ferguson, N. M., Fraser, C., Cauchemez, S., 2013. A new framework and software to estimate time-varying reproduction numbers during epidemics. American Journal of Epidemiology 178 (9), 1505–1512.

Ferguson, N. M., Laydon, D., Nedjati-Gilani, G., Imai, N., Ainslie, K., Baguelin, M., Bhatia, S., Boonyasiri, A., Cucunubá, Z., Cuomo-Dannenburg, G., Dighe, A., Fu, H., Gaythorpe, K., Green, W., Hamlet, A., Hinsley, W., Okell, L. C., Van, S., Thompson, H., Verity, R., Volz, E., Wang, H., Wang, Y., Walker, P. G. T., Walters, C., Winskill, P., Whittaker, C., Donnelly, C. A., Riley, S., Ghani, A. C., 2020. Impact of non-pharmaceutical interventions (NPIs) to reduce COVID-19 mortality and healthcare demand. Imperial College London (March).

Flaxman, S., Mishra, S., Gandy, A., Unwin, J. T., Coupland, H., Mellan, T. A., Zhu, H., Berah, T., Eaton, J. W., Guzman, P. N. P., Schmit, N., Cilloni, L., Ainslie, K. E. C., Baguelin, M., Blake, I., Boonyasiri, A., Boyd, O., Cattarino, L., Ciavarella, C., Cooper, L., Cucunubá, Z., Cuomo-Dannenburg, G., Dighe, A., Djaafara, B., Dorigatti, I., Van Elsland, S., Fitzjohn, R., Fu, H., Gaythorpe, K., Geidelberg, L., Grassly, N., Green, W., Hallett, T., Hamlet, A., Hinsley, W., Jeffrey, B., Jorgensen, D., Knock, E., Laydon, D., Nedjati-Gilani, G., Nouvellet, P., Parag, K., Siveroni, I., Thompson, H., Verity, R., Volz, E., Gt Walker, P., Walters, C., Wang, H., Wang, Y., Watson, O., Xi, X., Winskill, P., Whittaker, C., Ghani, A., Donnelly, C. A., Riley, S., Okell, L. C., Vollmer, M. A. C., Ferguson, N. M., Bhatt, S., 2020. Estimating the number of infections and the impact of non-pharmaceutical interventions on COVID-19 in 11 European countries. https://doi.org/10.25561/77731.

Gelman, A., Lee, D., Guo, J., 2015. Stan: A probabilistic programming language for bayesian inference and optimization. Journal of Educational and Behavioral Statistics 40 (5), 530–543.

Google, 2020. Google COVID-19 Community Mobility Reports. https://www.google.com/covid19/mobility/.

Hortaçsu, A., Liu, J., Schwieg, T., 2020. Estimating the fraction of unreported infections in epidemics with a known epicenter: An application to COVID-19. Working paper.

Lauer, S. A., Grantz, K. H., Bi, Q., Jones, F. K., Zheng, Q., Meredith, H. R., Azman, A. S., Reich, N. G., Lessler, J., 2020. The incubation period of coronavirus disease 2019 (COVID-19) from publicly reported confirmed cases: Estimation and application. Annals of Internal Medicine.

Muth, J. F., 1960. Optimal properties of exponentially weighted forecasts. Journal of the American Statistical Association 55 (290), 299–306.

Park, M., Cook, A. R., Lim, J. T., Sun, Y., Dickens, B. L., 2020. A systematic review of COVID-19 epidemiology based on current evidence. Journal of Clinical Medicine 9 (4), 967.

Shephard, N., 2015. Martingale unobserved component models. In: Koopman, S. J., Shephard, N. (Eds.), Unobserved Components and Time Series Econometrics. Oxford University Press, Oxford, pp. 218–249.

Thompson, R. N., Stockwin, J. E., van Gaalen, R. D., Polonsky, J. A., Kamvar, Z. N., Demarsh, P. A., Dahlqwist, E., Li, S., Miguel, E., Jombart, T., Lessler, J., Cauchemez, S., Cori, A., 2019. Improved inference of time-varying reproduction numbers during infectious disease outbreaks. Epidemics 29 (March).

Verity, R., Okell, L. C., Dorigatti, I., Winskill, P., Whittaker, C., Imai, N., Cuomo-dannenburg, G., Thompson, H., Walker, P. G. T., Fu, H., Dighe, A., Griffin, J. T., Baguelin, M., Bhatia, S., Boonyasiri, A., Cori, A., Cucunubá, Z., Fitzjohn, R., Gaythorpe, K., Green, W., Hamlet, A., Hinsley, W., Laydon, D., Nedjati-gilani, G., Riley, S., Elsland, S. V., Volz, E., Wang, H., Wang, Y., Xi, X., Donnelly, C. A., Ghani, A. C., Ferguson, N. M., 2020. Estimates of the severity of coronavirus disease 2019: A model-based analysis. Lancet Infectious Diseases 3099 (20), 1–9.

Wang, H., Wang, Z., Dong, Y., Chang, R., Xu, C., Yu, X., Zhang, S., Tsamlag, L., Shang, M., Huang, J., Wang, Y., Xu, G., Shen, T., Zhang, X., Cai, Y., 2020. Phase-adjusted estimation of the number of coronavirus disease 2019 cases in Wuhan, China. Cell Discovery 6 (1), 4–11.
