# Supplementary material for: Tracking ℛ of COVID-19: A new real-time estimation using the Kalman filter
Source: PLoS One. 2021 Jan 13;16(1):e0244474. doi: 10.1371/journal.pone.0244474 (PMC7806155; doi:10.1371/journal.pone.0244474)
Supplement: Attachment — Submitted filename: response_to_Reviewer.pdf [file pone.0244474.s003.pdf]

Dear Referee,

We are very grateful for your insightful and constructive suggestions on how to improve our paper, entitled "Tracking *R* of COVID-19: A New Real-Time Estimation Using the Kalman Filter."

All comments raised by you and the Editor have been addressed in the revised manuscript. Most suggestions have been incorporated in the main body, while a few are addressed in the Supplementary Appendix to avoid overextending the main paper.

When revising the paper, we re-ran our estimations using the most recent data vintage from the John Hopkins COVID-19 database. While doing so, we kept the same sample period as in our original submission, with the sample ending on May 6, 2020, to stay as close as possible to the initial manuscript. (Readers who wish to see the most up-to-date estimates can do so by visiting the online dashboard which is linked to in the abstract of the paper.) However, some data in the John Hopkins database has been retroactively adjusted since our first submission. Hence, even though we have kept exactly the same sample period, some estimates in the revised version of the paper differ slightly from the estimates in the original submission. However, all conclusions remain unchanged.

Point-by-point responses are provided below.

Dr. Francisco Arroyo-Marioli
Francisco Bullano,
Dr. Simas Kučinskas,
Dr. Carlos Rondón-Moreno

Comments by the Editor

1. *Please include a completed GATHER checklist (http://gather-statement.org/) and include in your supplementary material as well as make reference to this at the beginning of your methods section.*

We have included the GATHER checklist in the Supplementary Appendix (Section A.15) and added a reference in the methods section (lines 176–177).

2. *My understanding for COVID-19, is that a SEIR model should be used instead of the SIR. The authors refer to the SEIR formulation in the supplementary as part of their sensitivity analyses but I wonder why the main results were not just based on the SEIR assuming duration of infectiousness based on a published estimates/uncertainty range?*

We are grateful for this insightful comment. In short, we prefer the SIR specification because (i) simulations suggest that the estimates remain accurate even when the true model is SEIR rather than SIR; and (ii) we believe that the SIR structure is likely to produce more reliable estimates in practice, for reasons detailed below.

To respond to this comment, we have added the following paragraph to the conclusions section, motivating our choice of the SIR model (lines 408–425):

> Our estimates of R for COVID-19 are based on a structural relationship derived from the SIR model. By using the SIR model, we omit some features of the disease that are likely important when modeling its spread. In particular, the SIR model abstracts away from incubation periods as well as transmission during the incubation period. Nevertheless, we prefer the SIR specification, for two reasons. First, in simulations, we find that our estimator produces accurate estimates even when the true model is SEIR rather than SIR, as we show in S1 Appendix (Section A.5). Second, we believe that the SIR model is likely to produce more reliable estimates in practice. To use the SEIR model, the researcher would first

need to estimate the number of currently exposed individuals. Doing so would triple the number of model parameters. In particular, the researcher would additionally need to calibrate the (i) average duration of the incubation period (κ^{-1}); and (ii) relative infectiousness of exposed and infectious individuals (ε); see S1 Appendix (Section A.5) for details. While κ is arguably constant across countries and over time, ε is unlikely to be fixed. For example, greater mask usage is likely to reduce ε by differentially affecting transmission by symptomatic and pre- or asymptomatic individuals. Allowing for such time variation in ε, in addition to time-varying transmission rates (β_t), is challenging. That said, ideas in this paper can also be applied to models that are richer than the SIR model. Doing so may be an interesting avenue for future research.

3. *Discussion: please include more comparison/contrast with other similar published reproductive number estimates available in the literature.*

We thank the Editor for this valuable suggestion. In the revised version of the manuscript, we now include an empirical comparison to the Cori et al (2013) method, as implemented in the popular *EpiEstim* package in R. This exercise is included in the Supplementary Appendix (Section A.12). We find that the two sets of estimates are highly positively correlated empirically, with an average correlation coefficient (across countries) of 0.80, and a median correlation coefficient of 0.89.

In addition, we added a new paragraph to the discussion section that summarizes the pros and cons of our approach and discusses the empirical comparison to the Cori et al (2013) method (lines 426–438):

> Relative to existing methods for estimating *R*, we combine basic epidemiological theory with standard time-series filtering techniques, particularly Kalman filtering. This approach leads to a transparent closed-form estimator. The simplicity of the estimator allows us to study some of its properties analytically (e.g., the effects of potential data problems). Differently from most existing

approaches, our method can be applied using both Bayesian and frequentist techniques, and it does not require any tuning parameters beyond specifying the average serial interval. On the other hand, relative to less structural approaches such as that of Cori et al [3], our estimator may be more sensitive to potential model misspecification. Empirically, we find that our estimates and estimates obtained by the Cori et al method are highly positively correlated (average correlation: 0.80). However, the correlations are not perfect, suggesting that there is value in combining both estimators when tracking infectious diseases. Hence, our methodology brings an additional instrument to the researcher's toolbox.

Comments by the Referee

1. *Abstract: "the method is very easy to apply in practice" – consider being more specific about what makes it easy to apply.*

We thank the Referee for this point. We have changed the relevant sentence in the abstract as follows to highlight the fact that the method is easy to implement in practice using standard statistical software:

> The method is easy to implement in standard statistical software, and it performs well even when the number of infected individuals is imperfectly measured, or the infection does not follow the SIR model.

2. *Repeated throughout - when referencing material in the SI, the authors should include the section number and figure number to help the reader quickly locate the material.*

We have adjusted the text accordingly. We thank the reviewer for mentioning this.

3. *Line 2. At the beginning, the authors should stress the difference between R0 and effective reproduction number and include references for standard definitions. This*

difference is highlighted in Lines 82 through 84. However, it would be better to move this up in the introduction to give a better background.

We are grateful for this suggestion that substantially improves the readability and flow of the paper. We have adjusted the first paragraph of the paper to stress the difference between the two definitions. The updated paragraph now reads as follows (lines 2–10):

> The effective reproduction number (*R*) plays a central role in the epidemiology of infectious diseases. *R* is defined as the average number of secondary cases produced by a primary case [1–3]. The effective reproduction number varies over time, due to the depletion of susceptible individuals as well as changes in other factors, including control measures, contact rates, and climatic conditions. The basic reproduction number, denoted by R_0, measures the average number of secondary cases produced by a primary case when the population is fully susceptible [4, 5]. Analogously to the effective reproduction number, the basic reproduction number is also affected by multiple variables [6].

4. *Lines 36 to 39. References that support each of the approaches outlined should be cited here.*

We have added the necessary references supporting each of these two approaches (lines 44 and 46). We have also added a reference to a preprint by Gostic et al (2020) that makes a similar distinction between these two approaches to estimating *R* (line 43).

5. *Line 60. It would be good to be more specific/formal about what is meant by "well-behaved estimates"*

We have adjusted the manuscript to be more specific about what we had in mind, namely that the state-space approach automatically ensures that the estimates are temporally connected and not too "jagged." This feature is especially important if the estimates are to be used for informing the use of control measures. The adjusted sentence is now as follows (lines 66–68):

> Explicitly accounting for the dynamics in R via the state equation ensures that the estimated effective reproduction numbers are not excessively volatile, with the optimal amount of filtering estimated from the data.

6. *Lines 112. "As is well known, the local-level model is sufficiently flexible to capture rich dynamic patterns in the data." The authors should give some references here to support this assertion to help readers.*

We thank the Referee for this point. We have added references to two textbook treatments of the Kalman filter that discuss the local-level model in detail and provide a number of empirical applications. We have also slightly rewritten the relevant sentence to avoid overgeneralizing the applicability of the local-level model (lines 120–121).

7. *Line 160. A sentence about the sensitivity of your results to your chosen serial would be good here to summarise what you show in the supplementary (particularly as you note show in the SI that having an accurate estimate for the this parameter is required for your estimator to be robust to model misspecifications)*

We are grateful for this suggestion. In response to this comment we have added the following sentences that discuss how our estimates depend on the serial interval, including a small numerical example (lines 170–175):

> In general, by virtue of Eq (3), changing γ tilts the estimates of R_t around one, with higher values of the serial interval pushing the estimates away from one and lower values pushing the estimates towards one. For example, if R_t=1.5 for γ^{-1}=7 days, increasing the serial interval to 8 days increases the estimate to R_t≈1.57. Conversely, if R_t=0.5 for γ^{-1}=7 days, increasing the serial interval to 8 days decreases the estimate to R_t≈0.43.

8. *Line 168 & Figure 1. Is it meaningful to present Rt for the whole world aggregated together? Might be better to present more of your country level estimates.*

We agree with the Referee that estimates of R_t for the whole world can be difficult to interpret. For example, this "global" R_t may be flat because R_t is increasing in some countries while decreasing in others.

Following the suggestion of the Referee, we now plot our estimates for three additional countries (Brazil, India, and Germany), next to the countries already shown previously (China, US, and Italy). Our choice of the countries was motivated by a desire to (i) include important outbreaks of COVID-19; and (ii) capture a large fraction of the world's population. Combined, the six countries represent around 45% of the world's population. Our estimates for the remaining countries can be accessed in the online dashboard.

We have also updated the text discussing Figure 1 to reflect these changes (lines 191–227).

9. *Figure 2. Since different colours are used to denote Rt estimates for China, Italy and the US, there is no need to also use different linetypes.*

We understand and appreciate the comment made by the Referee. However, we are aware that it is common for many readers to print papers in black and white. In addition, some readers may be colorblind. In such cases, linetypes help the readers understand the figure quickly. We would therefore like to keep the current linetypes to ensure that all readers can easily grasp the graph.

10. *Line 181. Add reference to the Figure number in the SI.*

We have adjusted the reference as suggested (line 192).

11. *Line 187. "..but at a slower rate than previously observed in China". Can the authors quantify this rate? For example, the time difference between the start of the epidemic (as defined in this paper) and the first time point estimate of R is below 1?*

We thank the Referee for this very useful suggestion. In the updated version, we now quantify the time (in days since the start of the epidemic) it took for the point estimates of R_t to first fall below one (lines 196, 206, 212, and 218). In particular, it took 24 days for R_t to fall below one in China, while it took 36 days in Italy.

12. Line 195. The statement will be strengthened if the authors provide the estimate with confidence bounds. A similar comment applies when qualitative statements are made e.g., "somewhat", "almost" etc.

We have adjusted the sentence accordingly, and it now reads as follows (line 215):

> The point estimate of R_t in the US at the end of the sample is below one and equal to 0.92 (65% CI: 0.57–1.27).

In addition, we have also other qualitative statements to make them precise whenever possible.

13. Line 208. Add reference to the exact section number and figure that the reader should check.

We have added the reference as suggested (line 254).

14. Line 236. "Event study analysis" I do not think that this is a commonly used terminology in epidemiology, and the authors should provide a brief definition to help the reader.

We are grateful to the Referee for pointing out this issue. We now include a reference to a standard source on event studies in economics and finance and also provide a brief description of the methodology, including its potential problems (lines 284–293).

16. Line 238. Present 95% confidence intervals alongside these point estimates.

We have added these confidence intervals. The relevant sentence now reads as follows (lines 297–298):

> R_t declines substantially after a lockdown is introduced, going from 2.11 (95% CI: 1.84–2.38) on the day of the intervention to 0.99 (95% CI: 0.87–1.11) three weeks later.

17. Line 239. "There is no visually detectible break in the slope of R around the introduction of lockdown". Can the authors quantify what they mean by "around the time of lockdown"? Any change in policy or behaviour is not expected to have an immediate effect on R, and this should be taken into consideration. This comment also applies to Line 247 where the authors state that "R_t appears to be unaffected by lockdown." Consider rephrasing this. No counterfactual is available, and just because behaviour changed before lockdown doesn't mean it would have remained changed in the absence of a lockdown as you later mention. In addition, peoples initial behavioural change may have been influenced by discourse around potential upcoming lockdown and lockdowns introduced in other countries prior to introduction in their own country.

We thank the Referee for this important comment. In the revised version, we now specifically state that we study the behavior of R_t one week before and three weeks after the introduction of control measures (lines 294–295). We have also rephrased the statement in line 247 of the previous draft, so that it now reads as follows (lines 307–308):

> To further investigate the behavior of R_t in the four-week window around lockdowns, we use mobility data from Google's "COVID-19 Community Mobility Reports" [24].

18. Line 247. A brief description of the construction of the mobility index should be included in the main text. Can the authors check also that the decline in mobility before lockdown

is consistent across all countries considered, and for other combinations e.g. countries in Asia vs countries in Europe? Finally, my comment about qualitative statements is applicable to the description in SI ("the first principal component explains a little less than 85% of the total variance in the data"). Perhaps state the actual variance explained?

We appreciate this comment by the Referee, as that was indeed an oversight on our behalf. We have now added a paragraph explaining the construction of the mobility index and state the precise percentage of total variance explained by the first principal component. The relevant part reads as follows (lines 309–314):

> Google uses smartphone location data to measure changes in mobility (relative to pre-pandemic levels) for six six types of places: (i) groceries and pharmacies; (ii) parks; (iii) transit stations; (iv) retail and recreation; (v) residential; and (vi) workplaces. Since these measures are strongly correlated, we take the first principal component of the six time series to construct an overall mobility index. The first principal component explains 83.03% of the total variation in Google's mobility data.

It is more challenging to address the other part of the Referee's comment, namely, whether the behavior of mobility before the introduction of lockdown measures is similar in other countries. We cannot answer this question with the datasets currently used in our paper, as the dates of interventions are only available for the 14 European countries studied by Flaxman et al (2020). In addition, Google's mobility reports are not available for all countries globally.

Given these complications, and the fact that our paper is already quite long and has an extensive Supplementary Appendix, we have decided to leave a careful examination of this question for future work.

However, to at least partially address the Referee's concerns, we have performed a preliminary analysis. First, we used data from the Oxford COVID-19 Government Response Tracker

(OxCGRT) Index to obtain dates of lockdowns for countries across the world. We define the introduction date of a lockdown as the first date on which the C6 subcomponent of the OxCGRT Index takes a value of 2 or higher, meaning that the COVID-19 control measures "*require not leaving house with exceptions for daily exercise, grocery shopping, and 'essential' trips*" or "*require not leaving house with minimal exceptions (eg allowed to leave once a week, or only one person can leave at a time, etc)*" (link to OxCGRT codebook). To be consistent with the sample period studied in the paper, we only considered data up to May 6, 2020.

Overall, after removing the 14 Western European countries already studied in the paper, and imposing requirements on non-missing data for mobility data from Google and estimates of R, we are left with 22 countries that introduced lockdowns according to the definition above (Australia, Chile, Egypt, Estonia, India, Indonesia, Ireland, Israel, Kuwait, Mexico, Oman, Pakistan, Panama, Poland, Romania, Saudi Arabia, Singapore, Slovakia, South Africa, South Korea, Uruguay, Vietnam).

The picture below shows the behavior of the mobility index for these countries in the four-week window around the introduction of lockdowns. The observed pattern is very similar to that seen for the 14 Western European countries studied in the manuscript. While we acknowledge that the present analysis is very preliminary, it appears that the mobility patterns seen in Western Europe generalize to other countries as well.

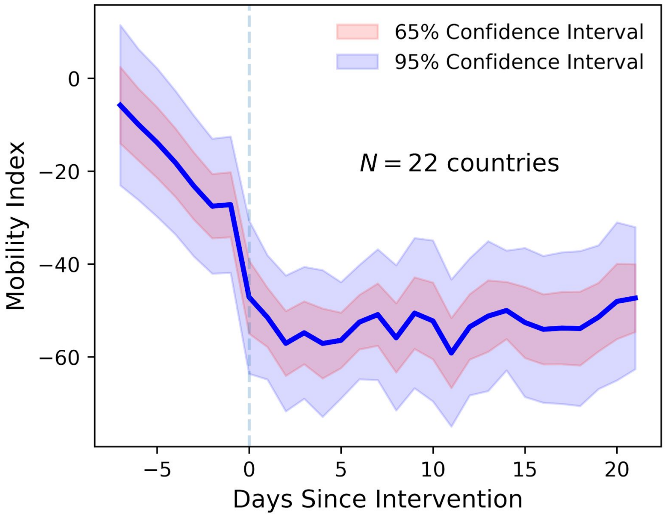

Line 266. State what is u_{i, t}.

The term u_{i,t} denotes the error term of the regression. We have rephrased the statement in line 266 of the previous draft, so that it now reads as follows (lines 332–334):

> [...] where $u_{i,t}$ denotes the stochastic error term of the regression. The term $NPI^{j}_{i,t}$ is an indicator variable that equals 1 after the *j*-th NPI is introduced, and zero before its introduction. The index *i* denotes countries, and *t* stands for the number of days since the outbreak of the epidemic.

19. Line 276. The only reason Rt can fall is because of... omit "why"

We have adjusted the sentence as suggested (line 343).

20. Table 2. If you could add concise titles to the columns in place of (1) to (4) it would be more immediately clear to the reader.

We thank the reviewer for this suggestion. In response, we have added a label describing the dependent variable of the regressions to make the table more self explanatory ("Dependent Variable: $log(R_t)$"). Each of the columns denotes a separate model and has an explicit reference in the text. The checkmarks in the bottom part of the table describe the model specifications. If possible, we would like to keep the current labels to avoid more clutter.

21. Line 312. Give a brief definition/detail on what is meant by endogeneity problems or use a different phrase – it is not guaranteed to be terminology that readers will have come across, though they will be familiar with the problem itself.

We are grateful to the Referee for pointing out this issue. By "endogeneity issues" we were referring to statistical problems that arise when a confounding variable is unobservable and therefore omitted from the analysis (omitted-variable bias), or when the dependent and independent variables are codetermined (simultaneity bias). For clarity, we have modified the

paragraph as follows, avoiding the use of the term "endogeneity issues" altogether (lines 378–389):

> As a result, even our best estimates might still suffer from statistical issues such as unobservable confounding variables or simultaneity bias. In particular, the timing of NPIs is not random. Countries that introduced NPIs earlier likely did so because they had previously observed a stubbornly high R_t. In that case, the dependent and independent variables would be simultaneously determined, yielding biased estimates. Moreover, since we cannot directly observe peoples' attitudes towards COVID-19 or government policies, we cannot control for other variables affecting human behavior. These potential issues notwithstanding, we find that people adjusted their mobility patterns *before* the introduction of lockdowns. We believe that these findings bolster the importance of accounting for changes in human behavior when evaluating the effects of NPIs.

We have also removed references to "endogeneity" in the rest of the paper.

Comments by the Referee on the Supplementary Appendix

22. Page 1 sentence one "also obtains" should be "is also obtained"

We have adjusted the sentence as suggested.

23. SI Section 6 page 8. This section is very interesting – inconsistent levels of reporting is a major issue in Rt estimation. I would make sure readers are clearly signposted to this section from the main text when you talk about data problems there.

We thank the Referee for this suggestion as well as the encouraging words. We have added a new paragraph discussing the implications of inconsistent levels of reporting to the section that presents our estimates for COVID-19. The new paragraph reads as follows (lines 228–240):

A natural concern with any estimator of R_t applied to COVID-19 is that the estimator may be biased if only a fraction of all COVID-19 cases is detected. In S1 Appendix (Section A.6), we study the performance of our estimator under various assumptions on the reporting of COVID-19 cases. We show analytically that our estimator remains exactly valid even when only a fraction of all cases is detected (e.g., 10% of all cases are detected), provided that the fraction of all cases detected is constant over time. The estimates are also accurate under some other cases of misreporting. However, if the fraction of detected COVID-19 cases changes a lot over short windows of time, the estimator is biased. Finally, we investigate the performance of our estimator in a number of additional cases of imperfect reporting (such as a ramp-up in testing) that may be important in practice using Monte Carlo simulations. Overall, we conclude that our estimator is robust to potential mismeasurement of COVID-19 cases in a number of empirically-relevant scenarios.

24. *SI page 18. "For both validation exercises performed in the present section, we include all countries for which we have at least 20 observations after the onset of the epidemic (100 cumulative cases of COVID-19 reached)." Can the authors state how many countries were included? Figure 4 states N =13, but I believe this means only the European countries considered minus one?*

We thank the Referee for bringing up this point, as the section indeed did not explain sample selection very clearly.

The Referee is absolutely correct that the validation exercises in the Supplementary Appendix include more countries than the 14 Western European countries studied in the section on non-pharmaceutical interventions. (To be clear, Figure 4 states $N = 13$ because Sweden never introduced a lockdown, and hence it is not part of the sample of countries used to construct Figure 4. We added an explanatory sentence to clarify this point (lines 295–296).)

In both validation exercises, we start with the original sample of 124 countries (i.e., the set of countries for which we have at least 20 daily observations available since the start of the epidemic). For the validation exercise using data on deaths, we additionally impose the restriction that the cumulative number of deaths has to be at least 50. This restriction leaves us with 270 observations (at the country-week level) across 68 countries. For the validation exercise using mobility data, we again start with the sample of 124 countries. However, Google does not provide mobility for all countries in this sample. Hence, we are finally left with 100 countries and 792 country-week observations.

We have clarified the discussion of the sample-selection procedures and added the exact number of (i) countries; and (ii) total number of observations for both validation exercises (Section A.8 of S1 Appendix).
